# Idelalisib: Practical Tools for Identifying and Managing Adverse Events in Clinical Practice

**DOI:** 10.6004/jadpro.2016.7.6.3

**Published:** 2016-09-01

**Authors:** Nancy Driscoll

**Affiliations:** North Shore-LIJ CLL Research and Treatment Center, New Hyde Park, New York

## Abstract

Idelalisib is a first-in-class oral selective inhibitor of phosphatidylinositol 3-kinase delta, which is selectively expressed in hematopoietic cells, where it is critical to B-cell receptor signaling and B-cell development and function. Idelalisib is approved in the United States for the treatment of relapsed chronic lymphocytic leukemia (CLL; in combination with rituximab), relapsed follicular lymphoma (FL), and small lymphocytic lymphoma (SLL) and in the European Union for the treatment of CLL (in combination with rituximab). Approval was based on clinical activity in a phase II trial in indolent non-Hodgkin lymphoma and a phase III trial in CLL. Because idelalisib is a relatively new treatment option for patients with relapsed CLL, SLL, and FL, with a safety profile distinct from other agents, it is important for advanced practitioners (APs) to familiarize themselves with the adverse event (AE) profile and educate their patients as well. As active members of the oncology care team, APs can play a vital role in optimizing outcomes in patients receiving idelalisib therapy. This review will familiarize APs with the AE profile of idelalisib and provide practical information about the identification and management of AEs associated with idelalisib therapy.

Non-Hodgkin lymphomas (NHLs) are a diverse group of malignancies, primarily of B-cell origin (Shankland, Armitage, & Hancock, 2012). The most common NHL subtypes include diffuse large B-cell lymphoma, chronic lymphocytic leukemia (CLL)/small lymphocytic lymphoma (SLL), multiple myeloma, and follicular lymphoma (FL; National Cancer Institute, 2014). In 2015, there were an estimated 71,850 new NHL diagnoses and 19,970 NHL-related deaths in the United States (Dupuis et al., 2015).

Introduction of the anti-CD20 monoclonal antibody rituximab (Rituxan) was an important treatment advance in NHL, and the National Comprehensive Cancer Network Clinical Practice Guidelines in Oncology (NCCN Guidelines) for NHL include rituximab monotherapy and combination immunotherapy regimens (NCCN, 2015). However, new therapies continue to emerge in an effort to address important unmet needs, including the need for effective and well-tolerated regimens for relapsed/refractory disease and for patients who are older and/or unfit. Newer agents in the NCCN Guidelines include the anti-CD20 monoclonal antibodies obinutuzumab (Gazyva) and ofatumumab (Arzerra) as well as the novel, targeted therapies ibrutinib (Imbruvica) and idelalisib (Zydelig).

## Background on Idelalisib

Idelalisib is a first-in-class oral selective phosphatidylinositol 3-kinase delta (PI3Kä) inhibitor (Lannutti et al., 2011). Activation of the PI3K pathway enhances the growth, survival, and metabolism of cancer cells (Engelman, 2009). PI3Kä, which is selectively expressed in hematopoietic cells (Chantry et al., 1997; Vanhaesebroeck et al., 1997), is critical to B-cell antigen-specific receptor (BCR) signaling as well as B-cell development and function (Jou et al., 2002). PI3Kä hyperactivation is observed in B-cell malignancies (Puri & Gold, 2012), and PI3Kä inhibition by idelalisib has been shown to induce apoptosis in malignant B-cell tumor lines (Lannutti et al., 2011). Idelalisib inhibits multiple signaling pathways (BCR, CXCR4, and CXCR5) involved in B-lymphocyte homing and retention and clonal expansion of normal and malignant B cells; thus, idelalisib impairs chemotaxis and migration and reduces cell viability (Hoellenriegel et al., 2011). The selective targeting of hematopoietic cells by idelalisib provides a potential mechanism of action for clinical activity, with low toxicity to vital organs compared with chemoimmunotherapy.

Idelalisib is approved by the US Food and Drug Administration (FDA) for relapsed CLL, in combination with rituximab, in patients for whom single-agent rituximab would be considered appropriate therapy owing to comorbidities (Gilead Sciences, 2014). It is also approved as monotherapy for relapsed FL or relapsed SLL in patients who have received at least two prior systemic therapies (Gilead Sciences, 2014).

Approval of idelalisib was based on the clinical activity and safety profile demonstrated in a phase II trial in indolent NHL (Gopal et al., 2014) and a phase III trial in CLL (Furman et al., 2014). In the European Union, idelalisib is indicated for combination therapy with rituximab for the treatment of CLL in patients who have received at least one prior treatment or as first-line treatment in patients with 17p deletion or *TP53* mutation unsuitable for chemoimmunotherapy (Fischer et al., 2012). It is also approved as a single agent for FL that is refractory to two previous lines of therapy (Fischer et al., 2012). Idelalisib provides a treatment option for patients who are less able to undergo standard chemotherapy.

## Role of the Advanced Practitioner

Rates of patient adherence to oral anticancer medications have been reported to vary widely, and adverse events (AEs) are a key factor in patient adherence and persistence (Ruddy, Mayer, & Partridge, 2009). Management of AEs is essential to limit potential treatment interruptions. Advanced practitioners (APs), including physician assistants and nurse practitioners, are an integral component of the oncology care team (Institute of Medicine, 2013; Levy, Gagnet, & Stewart, 2013) and play a key role in the identification and management of AEs.

In most states, APs can diagnose disease, order tests, make patient referrals, and prescribe medication (Christian, Dower, & O’Neil, 2007; Institute of Medicine, 2013). Many APs take on the role of primary care clinician for their patients (Institute of Medicine, 2013). A regional survey of physician assistants in oncology demonstrated extensive clinical responsibilities, including obtaining a patient history, performing physicals, making assessments, planning treatments, obtaining consent to treatment, and providing education (Ross, Polansky, Parker, & Palmer, 2010). A total of 77% of physician assistants reported they wrote chemotherapy orders. Over 60% of APs report ordering routine chemotherapy daily. This review is intended to provide APs with a practical guide to identifying and managing AEs associated with idelalisib therapy.

## Efficacy and Safety in Clinical Trials

**Efficacy**

The FDA approval of idelalisib was based on phase II and III efficacy and safety trials in indolent NHL (Gopal et al., 2014) and CLL (Furman et al., 2014), respectively. Efficacy results for these two trials are summarized in Table 1.

**Table 1:**
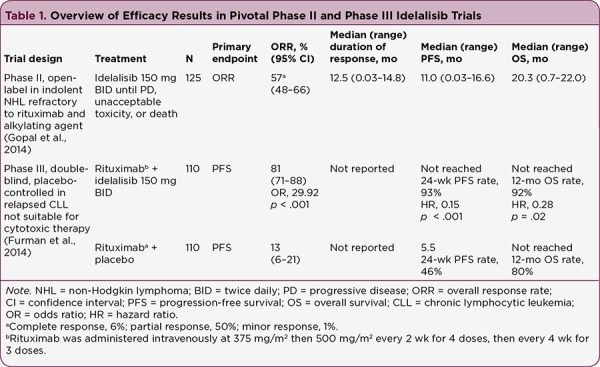
Overview of Efficacy Results in Pivotal Phase II and Phase III Idelalisib Trials

**Table 2:**
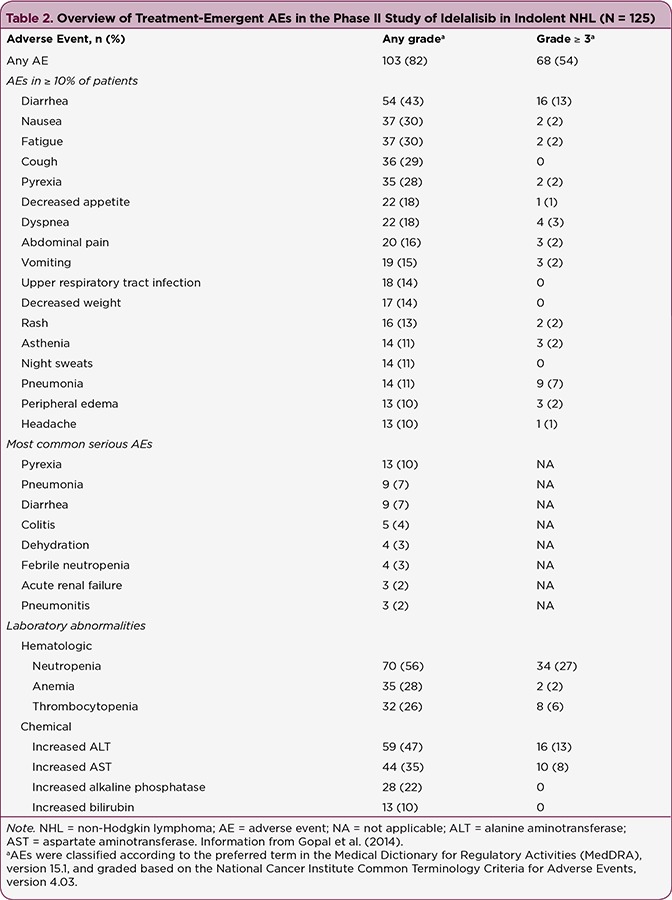
Overview of Treatment-Emergent AEs in the Phase II Study of Idelalisib in Indolent NHL (N = 125)

The phase II, open-label trial evaluated idelalisib at 150 mg twice daily (BID) in 125 patients with relapsed indolent NHL refractory to rituximab and an alkylating agent (Gopal et al., 2014). Subtypes of indolent NHL included FL (58%), SLL (22%), marginal zone lymphoma (12%), and lymphoplasmacytic lymphoma with or without Waldenström’s macroglobulinemia (WM, 8%). Patients were highly pretreated (median of four prior regimens), and 89% had stage III or IV disease.

The overall response rate (ORR) was 57% (95% confidence interval [CI], 48%–66%), with 7 complete responses (6%), 63 partial responses (50%), and 1 minor response (patient with WM). Responses were rapidly achieved (median time to response, 1.9 months) and durable (median duration of response, 12.5 months). Median progression-free survival (PFS) was 11.0 months, and median overall survival (OS) was 20.3 months, with an estimated 1-year survival of 80%.

The phase III randomized, double-blind, placebo-controlled study evaluated combination therapy with idelalisib at 150 mg BID or placebo plus rituximab (375 mg/m² followed by 500 mg/m² every 2 weeks for 4 doses, then every 4 weeks for 3 doses) in patients with relapsed CLL who were not candidates for cytotoxic chemotherapy because of clinically significant comorbidities, renal impairment, or previous therapy–induced myelosuppression (Furman et al., 2014). The study population comprised 220 patients, most of whom were ≥ 65 years of age and had advanced disease. Forty percent had at least moderate renal dysfunction, and 35% had poor bone marrow function. Patients had received a median of three prior regimens, including either an anti-CD20 monoclonal antibody or at least two cytotoxic chemotherapy regimens.

At the first prespecified interim analysis, the study was discontinued owing to overwhelming efficacy. At 24 weeks, the rate of PFS, the primary endpoint, was 93% in the idelalisib group vs. 46% in the placebo group (adjusted hazard ratio for disease progression or death, 0.15 [95% CI, 0.08–0.28]; *p* < .001). Median PFS was not reached in the idelalisib group and was 5.5 months in the placebo group. Patients in the idelalisib vs. placebo group had an improved ORR (81% vs. 13%; odds ratio, 29.92; *p* < 0.001) and improved 1-year rate of OS (92% vs. 80%; adjusted hazard ratio for death, 0.28 [95% CI, 0.09–0.86]; p = .02). Median OS was not reached in either group.

**Safety**

Tables 2 and 3 summarize treatment-emergent AEs in the phase II and phase III studies, respectively. The AEs of interest included diarrhea, colitis, pneumonitis, rash, transaminase elevations, and hematologic abnormalities (i.e., neutropenia, anemia, and thrombocytopenia). Common treatment-emergent AEs (≥ 10% of patients) were nausea, fatigue, diarrhea, and pyrexia. The most common grade ≥ 3 AEs were diarrhea, pneumonia, and dyspnea in the phase II study and diarrhea, pyrexia, and fatigue (idelalisib group) in the phase III study.

Rates of grade ≥ 3 neutropenia, thrombocytopenia, and anemia were 27%, 6%, and 2%, respectively, in the phase II study and 34%, 10%, and 5% in the phase III study idelalisib group, respectively. Rates of grade ≥ 3 alanine aminotransferase (ALT) and aspartate aminotransferase (AST) elevation were 13% and 8%, respectively, in the phase II study. In the phase III study, there was a 5% rate of ALT or AST elevation in the idelalisib group. The most common serious AEs in the two studies included pyrexia, pneumonia, diarrhea, febrile neutropenia, and pneumonitis.

In the phase II study, 25 patients (20%) discontinued treatment because of AEs, which were mainly transaminase elevation, diarrhea or colitis, and pneumonia or pneumonitis. In the phase III study, nine patients (8%) in the idelalisib group discontinued treatment because of AEs, which were mainly gastrointestinal and skin disorders. There was no overall increase in the rate of AEs with idelalisib vs. placebo, and there was a reduced rate of infusion-related toxicity (15% vs 28%).

In both studies, idelalisib toxicities were generally manageable with study drug interruption or dosage adjustment. In the phase II study, grade ≥ 3 diarrhea and/or colitis occurred in 20 patients (16%), with a median time to onset of 6 months (range, 1–13 months). Of them, six cases resolved without intervention or after dose reduction; six led to discontinuation of idelalisib; and eight resolved with dose interruption. Five patients who underwent dose interruption were able to resume treatment without recurrence of the toxicity.

Management of diarrhea/colitis was not detailed in the phase III study publication (Furman et al., 2014). In the phase II study, grade ≥ 3 transaminase elevations developed a median of 6.3 weeks (range, 4–11 weeks) after treatment onset; they were asymptomatic and uniformly resolved to grade ≤ 1 within a median of 3.9 weeks (range, 1–6 weeks) after treatment interruption. Ten of 14 patients who resumed treatment were able to continue with dose reduction and subsequent dose reescalation.

In the phase III study, grade ≥ 3 transaminase elevations occurred 8 to 16 weeks after treatment onset and in 4 of 6 cases (idelalisib group) resolved after treatment interruption, with successful treatment reinitiation. No patient withdrew from the study as a result of elevated transaminases. In the phase II study, one case of fatal pneumonitis was reported.

Overall, the AEs observed in these trials were consistent with those expected in patients with relapsed disease and extensive prior therapy. The observed safety profile was distinct from that of most other active agents for indolent NHL and CLL.

**Table 3:**
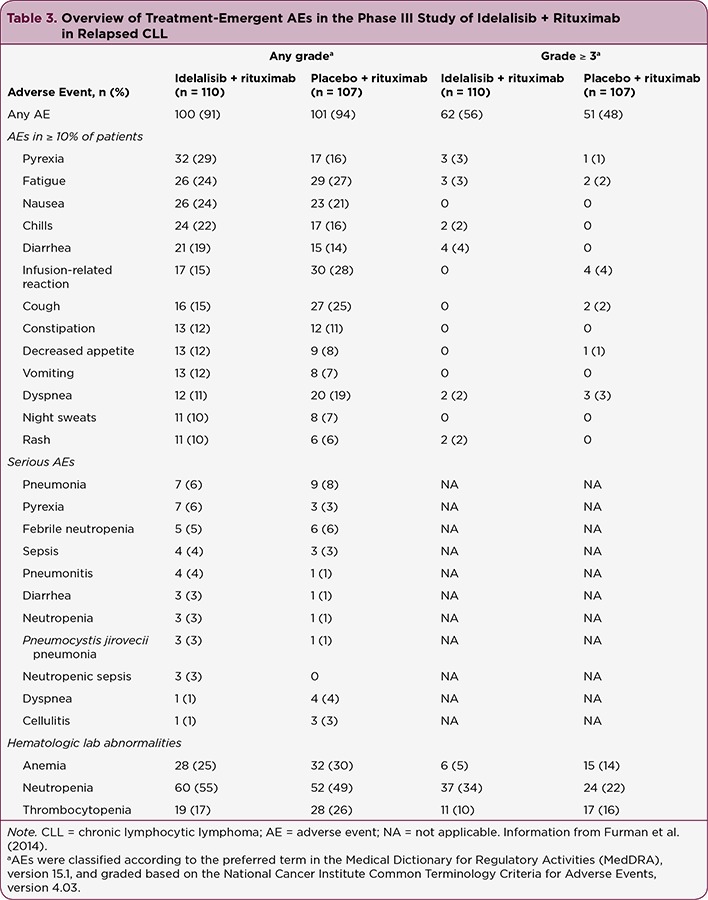
Overview of Treatment-Emergent AEs in the Phase III Study of Idelalisib + Rituximab in Relapsed CLL

## Identification and Management of Adverse events

**US Prescribing Information**

Recognizing and educating patients about the potential AEs associated with idelalisib are important to optimize treatment outcome. The safety information in the idelalisib US prescribing information (Gilead Sciences, 2014) provides a starting point for APs, including recommended patient education, monitoring, and dose modification (Table 4).

**Table 4:**
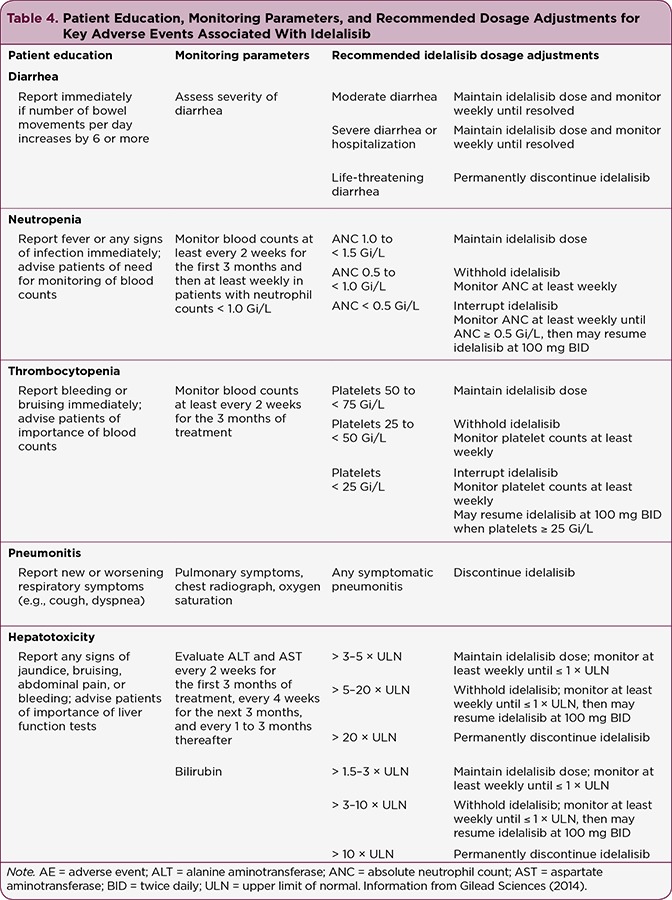
Patient Education, Monitoring Parameters, and Recommended Dosage Adjustments for Key Adverse Events Associated With Idelalisib

The idelalisib prescribing information carries a black box warning for hepatotoxicity, diarrhea/colitis, pneumonitis, and intestinal perforation. Accordingly, patients should be monitored for hepatic function, gastrointestinal and pulmonary symptoms, and bilateral interstitial infiltrates. In the event of severe hepatotoxicity or diarrhea/colitis, dose interruption is recommended until toxicity is resolved, with the option of subsequent rechallenge at a reduced dose (Table 4). For symptomatic suspected intestinal perforation, idelalisib should be discontinued.

The prescribing information also includes warnings and precautions for severe cutaneous reactions, anaphylaxis, and neutropenia, which also merit patient monitoring. Dose interruption is recommended in cases of severe neutropenia (Table 4). Idelalisib treatment is contraindicated in patients with a history of serious allergic reactions, including anaphylaxis and toxic epidermal necrolysis. Idelalisib is not advised during pregnancy because of the risk for embryofetal toxicity.

Idelalisib is metabolized to its major metabolite by aldehyde oxidase and cytochrome P450 3A (CYP3A; Gilead Sciences, 2014). In healthy subjects (n = 24), coadministration with rifampin, a strong CYP3A inducer, decreased idelalisib exposure by approximately 75%, and coadministration with idelalisib increased the exposure of midazolam, a CYP3A substrate, by 437% (Jin et al., 2015). In accordance with the U.S. prescribing information, it is advisable to avoid coadministration of idelalisib with strong CYP3A inducers (Gilead Sciences, 2014).

**Clinical Practice Experience**

Clinical practice experience with idelalisib provides an important context for the safety information provided in the prescribing information, which can be valuable in optimizing the time spent evaluating and managing AEs.

*Transaminitis*: To minimize the occurrence of transaminitis, it is best to avoid concomitant use of potentially hepatotoxic drugs. Elevations in ALT and AST often occur early in the treatment course (4–8 weeks) and are asymptomatic. Therefore, it is important for patients to come in for a visit and/or undergo laboratory tests every 1 to 2 weeks during that time. Patients with transaminase values one to five times the upper limit of normal (× ULN) can continue treatment if there is weekly monitoring. Patients with laboratory values 5 to 20 × ULN should undergo treatment interruption, to which ALT/AST elevations are generally responsive, with weekly monitoring until levels are < 3 × ULN. Treatment may then be resumed at the reduced dose of 100 mg BID. If transaminitis recurs, idelalisib should be discontinued.

Once transaminitis is diagnosed, APs should review current medications with patients to ensure that they are not taking potentially hepatotoxic agents. Questions should be specific because patients may not be aware of the potential hepatotoxicity of some over-the-counter medications, particularly acetaminophen, which is associated with transaminitis at doses of 4 g/day (Watkins et al., 2006). Furthermore, APs should ensure that patients have not had any significant increase in alcohol consumption while taking idelalisib.

*Diarrhea/colitis*: Diarrhea/colitis is one of the most frequent causes of idelalisib dose interruption or treatment discontinuation, and all cases merit close monitoring. Mild diarrhea (grade 1–2) commonly occurs early in the treatment course (first 1–2 months) and can typically be controlled with dietary modifications and antimotility treatments (e.g., loperamide). These mild cases do not require idelalisib dose interruption. Severe diarrhea often has a late onset (≥ 6 months). For any persistent grade 2 or 3 diarrhea, it is advisable to withhold idelalisib treatment and monitor closely.

Prompt use of steroids (e.g., budesonide, dexamethasone, prednisone) is helpful, but antimotility agents alone are ineffective (Coutré et al., 2015). Hospitalization may be necessary to manage dehydration and electrolyte abnormalities. Once diarrhea is resolved, idelalisib often may be resumed at a reduced dose (100 mg BID) without recurrence. Idelalisib should be permanently withdrawn in the instance of life-threatening diarrhea.

It is common for patients to underestimate or downplay diarrhea, reporting just a few daily episodes or attributing it to something they ate, then presenting with significant secondary weight loss, hypotension, and/or dehydration. To avoid such situations, it is essential to provide close monitoring and ongoing patient education from the time of treatment onset. Since late-onset diarrhea often coincides with prolonged visit intervals (2–3 months), it is important to regularly remind patients to report any diarrhea between visits.

*Pneumonitis*: In patients with underlying lung comorbidities (e.g., chronic obstructive pulmonary disease, interstitial lung disease), the risks and benefits of idelalisib therapy should be considered before deciding on treatment. Patients who present with pulmonary symptoms, including cough, dyspnea, interstitial infiltrates on chest radiograph, hypoxia, or a > 5% decrease in oxygen saturation, should be taken off idelalisib and evaluated for potential causes. Bronchoscopy is preferred. Providers may wish to initiate steroid treatment, with or without an antibiotic. Confirmed drug-induced pneumonitis, regardless of severity, requires permanent treatment discontinuation.

*Rash*: Patients receiving idelalisib have developed cutaneous reactions, including exfoliative dermatitis and various types of rash. Patients should be monitored for such reactions and treatment discontinued in the event of severe cases (Gilead Sciences, 2014). In our treatment center, we have had two patients with total-body, erythematous rashes, one of whom was diagnosed with psoriasis. Both patients discontinued idelalisib therapy.

*Bleeding events*: Bleeding events are uncommon with idelalisib. There is no contraindication to concomitant warfarin or antiplatelet therapy in the US prescribing information (Gilead Sciences, 2014). Nonetheless, the European prescribing information recommends monitoring of the international normalized ratio upon idelalisib coadministration with warfarin, dabigatran, or rivaroxaban because serum idelalisib concentrations may be increased (Fischer et al., 2012).

## Implications for Advanced Practitioners

An estimated 53% of physicians in the United States worked with APs in 2012 (Hing & Hsiao, 2014). In the oncology setting, an estimated 54% of U.S. oncologists worked with APs in 2007 (Erikson, Salsberg, Forte, Bruinooge, & Goldstein, 2007). A survey of Washington state medical oncologists determined that 68% were working with APs in 2008 (Britell, 2010). These numbers are likely to increase as the elderly population increases and places greater demand on the health-care system. Thus, APs are likely to have expanded opportunities to improve patient care (Erikson et al., 2007). An important component to improving patient outcomes is a proactive approach to patient monitoring as well as identification and management of AEs.

## Conclusion

Idelalisib is a relatively new treatment option for patients with relapsed CLL, SLL, and FL, with a safety profile distinct from other active agents. Most patients receiving idelalisib therapy will experience at least one AE during the course of treatment. By familiarizing themselves with the AE profile of idelalisib and educating patients about potential signs and symptoms, APs can help avoid unnecessary testing and delays in treating AEs for patients receiving idelalisib.

**Acknowledgment**

Editorial support for the preparation of this manuscript was provided by Amy Zannikos, PharmD, CMPP, and Nicole Strangman, PhD, of C4 MedSolutions, LLC, a CHC Group company (Yardley, PA), with funding from Gilead Sciences.
